# Deletion of *C1ql1* Causes Hearing Loss and Abnormal Auditory Nerve Fibers in the Mouse Cochlea

**DOI:** 10.3389/fncel.2021.713651

**Published:** 2021-08-25

**Authors:** Yue Qi, Wei Xiong, Shukui Yu, Zhengde Du, Tengfei Qu, Lu He, Wei Wei, Lingjun Zhang, Ke Liu, Yi Li, David Z. He, Shusheng Gong

**Affiliations:** ^1^Department of Otolaryngology Head and Neck Surgery, Beijing Friendship Hospital, Capital Medical University, Beijing, China; ^2^Department of Otology, Sheng Jing Hospital, China Medical University, Shenyang, China; ^3^Department of Otolaryngology Head and Neck Surgery, Beijing Tongren Hospital, Capital Medical University, Beijing, China; ^4^Department of Biomedical Sciences, Creighton University, Omaha, NE, United States

**Keywords:** C1QL1, hair cells, innervation, hearing loss, mice

## Abstract

Complement C1q Like 1 (C1QL1), a secreted component of C1Q-related protein, is known to play an important role in synaptic maturation, regulation, and maintenance in the central nervous system. *C1ql1* is expressed in adult cochlear inner and outer hair cells (IHCs and OHCs) with preferential expression in OHCs. We generated *C1ql1* null mice to examine the role of C1QL1 in the auditory periphery. *C1ql1*-null mice exhibited progressive hearing loss with elevated thresholds of auditory brainstem response and distortion product otoacoustic emission. Confocal microscopy showed that the number of nerve fibers innervating both IHCs and OHCs was significantly reduced. However, spiral ganglion neurons appeared to be normal under electron microscopy. IHC development and survival were not affected by deletion of *C1ql1.* Voltage-clamp recording and immunocytochmistry combined with confocal microscopy showed *C1ql1*-null IHCs showed no significant reduction of pre-synaptic proteins and synaptic vesicle release. This is in contrast to significant OHC loss in the KO mice. Our study suggests that *C1ql1* is essential for development of hair cell innervation and OHC survival. But maturation of presynaptic machinery in IHCs does not depend on C1QL1.

## Significance Statement

• We generated a *C1ql1* null mouse model and examined the role of C1QL1 in cochlear hair cells. The *C1ql1*-null mice had progressive loss of outer hair cells and hearing.
• We showed that the number of nerve fibers innervating both inner and outer hair cells was significantly reduced in *C1ql1*-null mice.
• We showed that *C1ql1* is essential for development of hair cell innervation and outer hair cells survival.
• Our study suggests *C1ql1* might be a deafness-related gene.

## Introduction

Hair cells of all vertebrates are polarized neuroepithelial cells that serve as the sensory receptors for the acoustical, vestibular, and lateral-line organs (Beurg et al., 2006; Salvi et al., 2016). Hair cells transduce mechanical stimuli into electrical activity (Hudspeth and Corey, 1977; Marcotti et al., 2014). Inner hair cells (IHCs) and outer hair cells (OHCs) are the two types of sensory receptor cells critical for hearing in the mammalian cochlea. IHCs and OHCs are different in morphology and function (Dallos, 1992; Li et al., 2019). IHCs, innervated by type I nerve fibers, are considered to be the true sensory receptor and transmit information to the brain. OHCs, innervated by type II fibers and predominantly by efferent fibers (Thiers et al., 2008; Huang et al., 2012), serve as the effector cell that boosts input to IHCs by a receptor potential-driven somatic motility (Brownell et al., 1985; Zheng et al., 2000; Liberman et al., 2002).

Several previous studies examined cell-specific transcriptomes of adult IHCs and OHCs to identify genes that are preferentially expressed since these genes are likely involved in defining unique morphology or function of the two cell types (Liu et al., 2014; Li et al., 2018; Ranum et al., 2019). Among many genes that are preferentially expressed, *C1ql1* is one of the top 10 most differentially expressed genes with 7.7 Log2 fold difference favoring OHCs (Liu et al., 2014; Li et al., 2018). C1q-like family members (*C1ql1*–*C1ql4*), all containing a specific gC1q domain and widely expressed in the brain (Iijima et al., 2010; Shimono et al., 2010), are implicated in synapse formation or elimination in the central nervous system (CNS) (Bolliger et al., 2011; Kakegawa et al., 2015; Sigoillot et al., 2015). C1QL1 can select and maintain dominant nerve fibers, induce pruning to eliminate excess nerve fibers, and this selective and pruning effect is present in both developing and adult animals (Kakegawa et al., 2015), especially in the motor neurons in cerebellum. Since *C1ql1* is highly expressed in OHCs, which serve as a motor element for cochlear amplification, we speculate that C1QL1 gene may play a key role in OHC function and survival as well as establishing and maintaining innervations in hair cells.

We generated *C1ql1* null mice to examine the role of C1QL1 in cochlear hair cells. We showed that *C1ql1*-null mice had progressive loss of OHCs and hearing. Confocal microscopy showed that the number of nerve fibers innervating both IHCs and OHCs was significantly reduced. Interestingly, no significant IHC and spiral ganglion neuron loss was observed. Voltage-clamp recording and immunocytochmistry combined with confocal microscopy showed *C1ql1*-null IHCs showed no significant reduction of pre-synaptic proteins and synaptic vesicle release. In contrast, significant OHC loss was observed in the KO mice. Our study suggests that *C1ql1* is essential for development of hair cell innervation and OHC survival. But maturation of presynaptic machinery in IHCs does not depend on C1QL1.

## Materials and Methods

### Ethics Statement

All animal experiments were performed with approval of Animal Care Committee of the Capital Medical University. All efforts were made to minimize animal suffering and the number of animals used.

### Generation of *C1ql1* Knockout Mice

Knockout (*C1ql1* KO) mice were generated on Kunming(KM)background mice. Mouse model with *C1ql1* KO was generated by CRISPR/Cas9 technique. The null mutation of mouse *C1ql1* was created by homologous recombination. To determine the nucleotide sequence of mutated alleles, mouse genomic DNA was tested by the following primers: *C1ql1* forward, 5′-CAGTGGCCCCAGCCAGGGAAGG-3′ and *C1ql1* reverse, 5′-AGGCGCACCGCTCTGCTCGCTC-3′. Newborn mice were examined by Sanger sequencing and the sequencing sample were taken from mice tail tip tissue. Mice of both sexes aged from postnatal day 0 to 6 months were used for the study.

### Auditory Brainstem Response (ABR) Measurements

Mice were tested under intraperitoneal anesthesia with ketamine (100mg/kg, Gutian Pharmaceutical Co., Ltd., Fujian, China) and xylazine (10mg/kg, Sigma-Aldrich Co. Llc., United States). An isothermal pad was used to keep body temperature of mice during audiometric testing. Needle electrodes and ground electrode were inserted subcutaneously beneath the pinna of the test ear, at the vertex and the contralateral ear, respectively. Auditory brainstem response (ABR) was recorded by Tucker-Davis Technologies (TDT) System III (Alachua, FL, United States). Both click (100 μs) and tone pips (4, 8, 12, 16, 32, and 40 kHz) were used for sound stimuli. 1024 responses were sampled and averaged at each sound level. Amplitude and latency of ABR wave I, and I–V wave interval was assessed at 90 dB SPL for each stimulus frequency. The lowest stimulus intensity that elicited a response was identified as ABR threshold, which was obtained by reducing the stimulus intensity in 10 dB SPL steps and then 5 dB steps. ABR threshold was determined by two trials to show repeatability.

### Distortion Product Otoacoustic Emission (DPOAE) Measurements

Distortion product otoacoustic emission responses were recorded by TDT System III. Mice were anesthetized as mentioned above and their external auditory canals were cleaned before DPOAE test. Two primary tones (f1 and f2) were presented with an f2/f1 ratio of 1.2. The emitted acoustic signal was set at 65 dB SPL ranging from 1k to 20 kHz. Input/output functions of DPOAE were calculated for each tested frequency. The threshold of DPOAE was defined as 6 dB SPL above the noise floor.

### Immunofluorescence

Mice were sacrificed by cervical dislocation and decapitated under anesthesia. Cochleae were removed and soaked in 0.01M phosphate-buffered saline (PBS) containing 4% paraformaldehyde (4% PFA/PBS) overnight at 4°C. Specimens were washed three times in PBS and subsequently treated with 10% EDTA decalcifying solution (PH = 7.2) for 12 h at room temperature (20–30°C). Then the cochlear tissues were carefully dissected under stereoscopic microscope (Nikon smz1000, Japan). Specimens were subsequently permeabilized by 0.3% Triton X-100 in 0.2 M PBS with 10% normal goat serum (ZSGB-BIO) for 2 h. Immunological histological chemistry (IHC) staining was performed using primary antibodies overnight at 4°C, followed by incubation with species-specific secondary antibodies for an additional 120 min at room temperature. Primary antibodies used were as follows: rabbit anti-C1QL1 polyclonal antibody (1:200, Bioss antibodies, bs-11045R), rabbit anti-myosin VIIa antibody (1:300, Proteus Biosciences, 25-6790), chicken anti-neurofilament 200 antibody (1:200, Chemicon, AB5539), and rat anti-myelin basic protein antibody (1:200, Millipore, MAB386). Fluorescent dye-conjugate secondary antibodies were conjugated to Alexa Fluor^TM^ 488, 568, or 647 (1:300, Invitrogen/Molecular Probes, Carlsbad, CA, catalog number: A11008, A21245, A11036, A11006, A21131, A21124, A11039, and A11041). Hair cell stereocilia were labeled with Alexa Fluor^TM^ 488 Phalloidin (Invitrogen/Molecular Probes, Carlsbad, CA, A12379). Prepared specimens were mounted on cover lip with antiblech mounting medium with DAPI (ZSGB-BIO, ZLI-9557).

### Laser Confocal Microscopy

Cochlea were imaged by a high-resolution confocal microscope (TCS SP8 II; Leica Microsystems, Wetzlar, Germany) with a 63 × oil-immersion lens. Optimal excitation wavelengths were 488 nm (green), 568 nm (red), or 647 nm (far-red). DAPI was observed under an excitation wavelength of 358 nm (blue). Sequence scanning in z-axis was performed with an interval of 0.5 μm/layer from top to bottom, and the images were then superimposed.

### Transmission Electron Microscopy (TEM)

For transmission electron microscopy (TEM),mice were sacrificed after anesthetized as mentioned above. The cochlear samples were removed and fixed overnight in 2.5% glutaraldehyde at 4°C. Specimens were rinsed with PBS in the next day, incubated in 1% osmium tetroxide for 2h at room temperature (20–30°C). Then specimens were dehydrated with graded acetone concentrations. Specimens were incubated in acetone mixed with Epon 812. Sections were cut at a 50nm thickness, located on copper grids, stained with uranyl acetate and lead citrate. Finally, the cochlear sections were examined in a transmission electron microscope (JEM-1400plus, JEOL, Japan). Images were captured with digital camera.

### Electrophysiological Experiments

Freshly dissected organ of Corti from the apical turn of *C1ql1* knockout and wildtype mice at postnatal days 15 to 30 were used for real time capacitance measurement under the voltage-clamp condition. The extracellular solution contained (in mM): NaCl 135; KCl 1.3; CaCl2 1.3; MgCl2 0.9; NaH2PO4 0.7; Glucose 5.6; Na pyruvate 2; HEPES 10, pH 7.4, 305 mOsm. An EPC10 triple amplifier controlled by pulse software Patchmaster (HEKA Elektronik, Germany) was used for recording. Patch pipettes were pulled with a micropipette Puller P-97 Flaming/Brown (Sutter Instrument, Novato, CA, United States) and coated with dental wax to minimize pipette capacitance. The resistance range of the electrode was between 4 and 5 MΩ. Patch pipettes were filled with an intracellular cesium-based solution containing (in mM): Cesium methanesulfonate 118;CsCl 10; HEPES 10; EGTA 1;TEA-Cl 10; MgATP 3 and NaGTP 0.5; pH 7.2, 310 mOsm.

Real-time capacitance measurements (Cm) were performed using the Lock-in amplifier Patchmaster software (HEKA) by applying a 1 kHz command sine wave (amplitude 20 mV) at holding potential (−80 mV) before and after the pulse experiment, Cells with a holding current exceeding 50 pA at −80 mV were excluded from analysis. The time interval between each depolarization was set at 20 seconds to allow full replenishment of the RRP. ΔCm was estimated as the difference of the mean Cm over 300 ms after the end of the depolarization (the initial 50 ms were skipped) and the mean prepulse capacitance (300 ms) dates were analyzed using IGOR Software.

### Statistical Analysis

Statistical analyses were performed using GraphPad Prism 7 software (GraphPad Software Inc, La Jolla, CA, United States) and IBM SPSS Statistics software. Means ± SEM (standard error of the mean) were calculated. Student’s *t* test was utilized to compare variables in two groups and one-way analysis of variance (ANOVA) was utilized to compare variables in multiple groups. Significant statistical differences were defined as ^∗^*P* < 0.05, ^∗∗^*P* < 0.01, and ^∗∗∗^*P* < 0.001.

## Results

### Expression of *C1ql1* in the Adult Cochlea and Loss of C1QL1 in Knockout Mice

**FIGURE 1:**
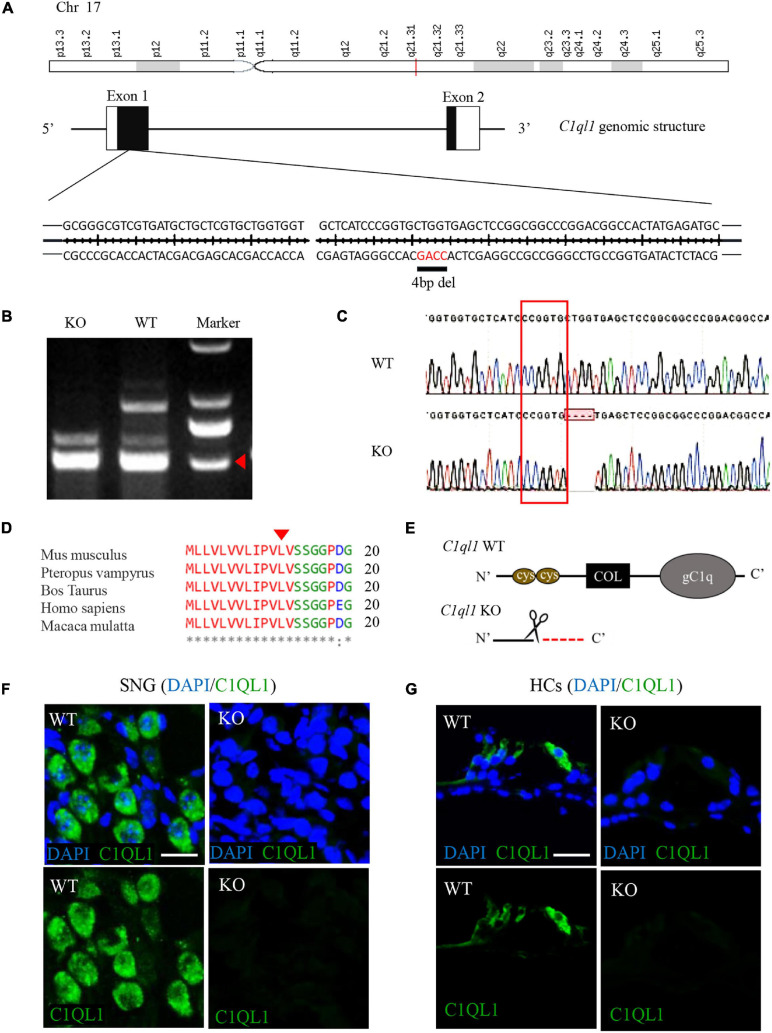
Construction of *C1ql1* knockout mice. **(A)** Mouse *C1ql1* gene located in Chr17 contains two exons, and a 4 base-pair (bp) deleted at nucleotide 34 (c.34_37delCTGG) in the exon 1 revealed *C1ql1* KO mice. **(B)** The different bands of *C1ql1* gene sequencing between *C1ql1* gene WT mice and *C1ql1* gene KO mice. **(C)** The DNA sequencing of *C1ql1* showed the c.34_37delCTGG mutation in *C1ql1* KO mice. **(D)** The c.34_37delCTGG mutation was located in the height region between species. **(E)** The critical domains of C1QL1, and c.34_37delCTGG mutation resulted in domain deletions. **(F)** C1QL1 labeled by anti-C1QL1 (green) were detected in spiral ganglion cell of *C1ql1* WT mice but not in *C1ql1* KO mice. Scale bar = 10 μm. **(G)** C1QL1 labeled by anti-C1QL1 (green) were also detected in hair cells of *C1ql1* WT mice but is missing in *C1ql1* KO mice. Scale bar = 10 μm. Nucleus of cells were marked with DAPI (blue) in **(F,G)**.

*C1ql1* was expressed in adult IHCs and OHCs based on RNA-seq and microarray analyses (Liu et al., 2014; Li et al., 2018). We used immunofluorescence staining to show where *C1ql1* is expressed in the organ of Corti in adult wild-type mice. As shown in Figures 1F,G, positive staining is seen in the cell bodies of both hair cells (HC) and spiral ganglion cells (SGC).

*C1ql1* belongs to C1q family with four known members (*C1ql1*, *C1ql2*, *C1ql3* and *C1ql4*). These members are highly homologous and all have a signature C1Q domain (Kishore and Reid, 2000). Mouse *C1ql1* gene in Chr17 contains two exons encoding 258 amino acids. We generated *C1ql1* KO mice by CRISPR/Cas9 genome editing technique. DNA sequencing in *C1ql1* KO mice revealed that a 4 base-pair (bp) fragment was deleted at nucleotide 34 (c.34_37delCTGG) of *C1ql1* gene, which caused a frameshift mutation and a premature appearance of termination codon (p. Leu12fsX1) in exon 1 (Figures 1A–C). This mutation was located in the conserved region between species (Figure 1D), and it was predicted that the mutation could lead to truncation of the C1QL1 protein and loss of the critical C1q domain (Figure 1E). We used immunofluorescence staining to confirm that C1QL1 was deleted by using an anti-C1QL1 polyclonal antibody. As shown in Figures 1F,G, the expression of C1QL1 is no longer detectable in *C1ql1* KO mice. This again confirms that *C1ql1* was successfully deleted.

### Mice With Defective *C1ql1* Gene Have a Significant Hearing Impairment

**FIGURE 2:**
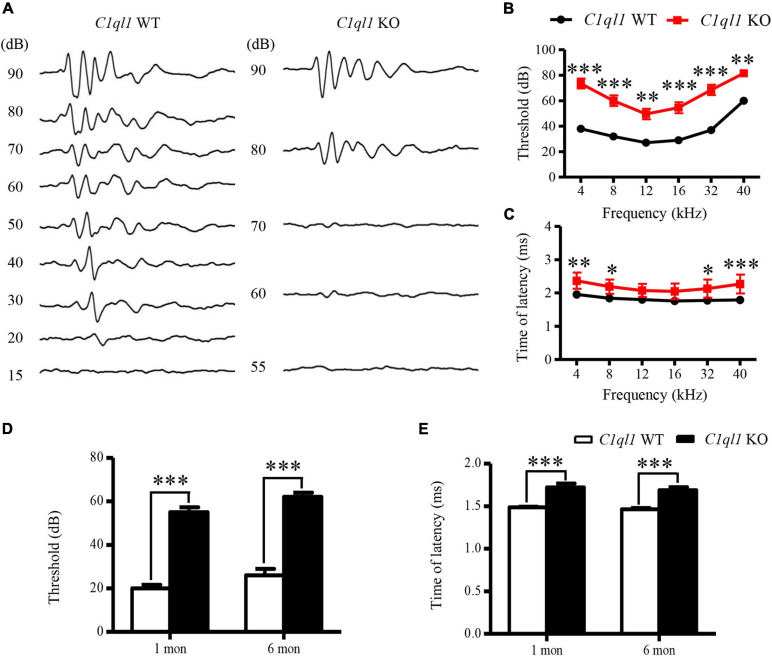
Changes of ABR in *C1ql1* KO mice. **(A)** Representative waves of click ABR in *C1ql1* WT mice and *C1ql1* KO mice. **(B)** Changes in threshold of ABR between *C1ql1* KO mice and *C1ql1* WT mice (*n* = 5). **(C)** Changes in latency of ABR wave I between *C1ql1* KO mice and *C1ql1* WT mice. **(D)** Statistical differences in click ABR thresholds between *C1ql1* KO mice and *C1ql1* WT mice in postnatal 1 and 6 months (*n* = 5). **(E)** Statistical differences in latency of click ABR wave I between *C1ql1* KO mice and *C1ql1* WT mice in postnatal 1 and 6 months (*n* = 5). ^∗^*P* < 0.05, ^∗∗^*P* < 0.01, and ^∗∗∗^*P* < 0.001.

Auditory function was evaluated by measuring ABR in *C1ql1* KO mice. Age-matched wild-type littermates were used for comparison. A 20 to 40 dB elevation of ABR threshold was observed in all frequencies (4, 8, 12, 16, 32, and 40 kHz) tested in the *C1ql1* KO mice in comparison to their wildtype littermates at 6 months (Figures 2A,B). The elevation in ABR thresholds of *C1ql1* KO (*n* = 5) and wild-type mice (*n* = 5) were statistically significant at all frequencies tested. Thresholds were also elevated when click-evoked ABR was used for measurements (Figure 2A).

We assessed the latency of wave I in the ABR waveforms. The latency is often used to evaluate the function of auditory nerve fibers. In *C1ql1* KO mice, the latency of wave I showed significant delays compared to those of the WT mice at 6 months (Figure 2C). Our data suggest that deletion of *C1ql1* led to deterioration of auditory function, in part by the abnormal signal transmission starting from cochlea. The differences in ABR threshold and latencies of wave I were detected from 1 month and to 6 months (Figures 2D,E).

**FIGURE 3:**
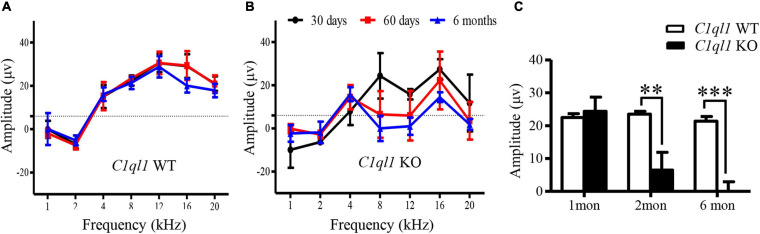
Changes of DPOAE in *C1ql1* KO mice. **(A,B)** DPOAE in *C1ql1* WT mice and *C1ql1* KO mice. **(C)** Statistical differences in DPOAE between *C1ql1* KO mice and *C1ql1* WT mice in postnatal 1, 2, and 6 months at 12 kHz (*n* = 4). Statistical significance was defined as *P < 0.05, ** *P* < 0.01, and ***P < 0.001.

We also examined outer hair cell (OHCs) function and status in the *C1ql1* KO mice. Unlike IHCs, we found significantly abnormal morphology and function of OHCs in the knockout mice. We measured the magnitude of distortion product evoked otoacoustic emissions (DPOAE) to assess function of OHCs. In the wild type mice, the amplitude of DPOAE did not change before 6 months (Figure 3A). In the *C1ql1* KO mice, the DPOAE magnitude was not significantly different from the wild type mice at 1 month (*n* = 4, *t* = 0.4257, *p* > 0.05). However, a progressive reduction of DPOAE amplitudes was observed in *C1ql1* KO mice after 2 months (*n* = 4, *t* = 3.823, p < 0.01) (Figure 3B). The reduction of DPOAE magnitude was significant between the *C1ql1* KO mice and wild types (*n* = 4, *t* = 4.782, p < 0.001) (Figure 3C).

### Absence of C1QL1 Caused Abnormal Auditory Nerve Fibers

**FIGURE 4:**
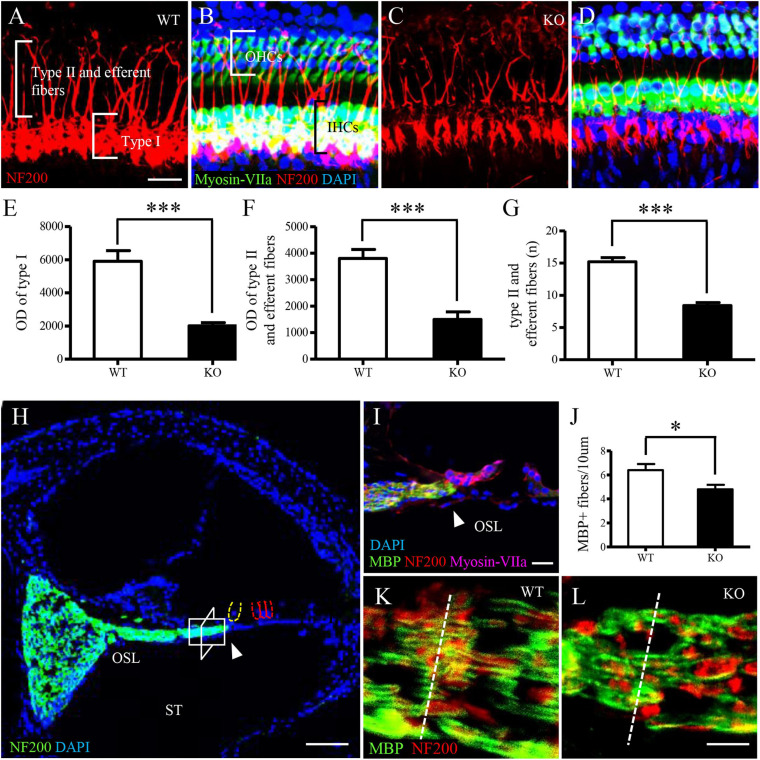
Auditory nerve fibers in *C1ql1* WT and *C1ql1* KO mice. **(A,C)** Type I, type II auditory nerve fibers and efferent fibers (red, labeled by anti-NF200) in *C1ql1* WT mice **(A)** and in *C1ql1* KO mice (C). Scale bar = 10 μm. **(B,D)** Morphology of cochlea under basilar membrane stretched preparation technique. **(B)** Cochlear basilar membrane in *C1ql1* WT mice. **(D)** Cochlear basilar membrane in *C1ql1* WT mice. Cytoplasm of hair cells was marked with anti-myosin VIIa antibody (green), nucleus of cells with DAPI (blue), and auditory nerve fibers and efferent fibers with anti-NF200 (red). Scale bar = 10 μm. **(E)** is the statistical difference in optical density (OD) value of Type I auditory nerve fibers and **(F)** is the statistical difference in OD value of Type II auditory nerve fibers and efferent fibers between *C1ql1* KO mice and *C1ql1* WT mice. **(G)** Is the statistical difference in number of Type II auditory nerve fibers and efferent fibers between *C1ql1* KO mice and *C1ql1* WT mice. **(H)** Morphology of cochlea under cochlear horizontal sections. Scale bar = 25 μm. **(I)** Amplification of osseous spiral lamina (OSL) regions in cochlea. Scale bar = 10 μm. The arrow points to habenular opening in **(H,I)**. **(J)** Statistical difference in number of Type I auditory nerve fibers between *C1ql1* KO mice and *C1ql1* WT mice. Measurements were made in the area about 20–30 mm from the habenular opening. The measured areas in *C1ql1* WT mice and *C1ql1* KO mice were showed in **(K,L)**, respectively. Scale bar = 5 μm. ^∗^*P* < 0.001 and ^∗∗∗^*P* < 0.001.

We investigated auditory nerve fibers and spiral ganglion cells (SGCs) in the *C1ql1* KO mice using immunofluorescence staining. Deletion of *C1ql1* affected the morphology of both type I, type II nerve fibers and efferent fibers (Figures 4A–D). We measured the diameter and number of auditory nerve fibers between WT mice and *C1ql1* KO mice when the fibers were stained with anti-NF200 antibodies. A 50 × 50 μm area was used for measuring diameter and number of type I auditory nerve fibers, type II auditory nerve fibers and efferent fibers. Figure 4 shows that the diameter of type (*P* = 0.0018), type II auditory nerve fibers and efferent fibers (*P* < 0.001) in *C1ql1* KO mice (*n* = 10) were significantly lower than those in WT littermate (*n* = 10) (Figures 4E,F). We measured the number of Type I nerve fibers and the nerve fibers passing through the tunnel which include both Type II and efferent fibers to OHCs. Type I auditory nerve fibers were counted in osseous spiral lamina regions on cochlear horizontal sections. Measurements were made in the area between habenular opening and spiral ganglia. The area measured was about 20–30 mm from the habenular opening in mice (Xing et al., 2012). As shown in Figure 4J, the number of type I auditory nerve fibers in the *C1ql1* KO mice (*n* = 5) is significantly lower than that of the WT mice (*P* = 0.0353, *n* = 5). The number of nerve fibers in the tunnel was also significantly reduced in adult *C1ql1* KO mice (15.20 ± 0.6464, *n* = 10) compared to that of the WT mice (8.400 ± 0.4761, *n* = 10). The number was counted in an area of 50 × 50 μm (Figure 4G). Our results indicate that deletion of *C1ql1* reduces both the type I auditory nerve fibers, type II auditory nerve fibers and efferent fibers innervating hair cells.

### Deletion of C1ql1 Did Not Affect Morphology of Myelin Basic Protein (MBP) and SGC

**FIGURE 5:**
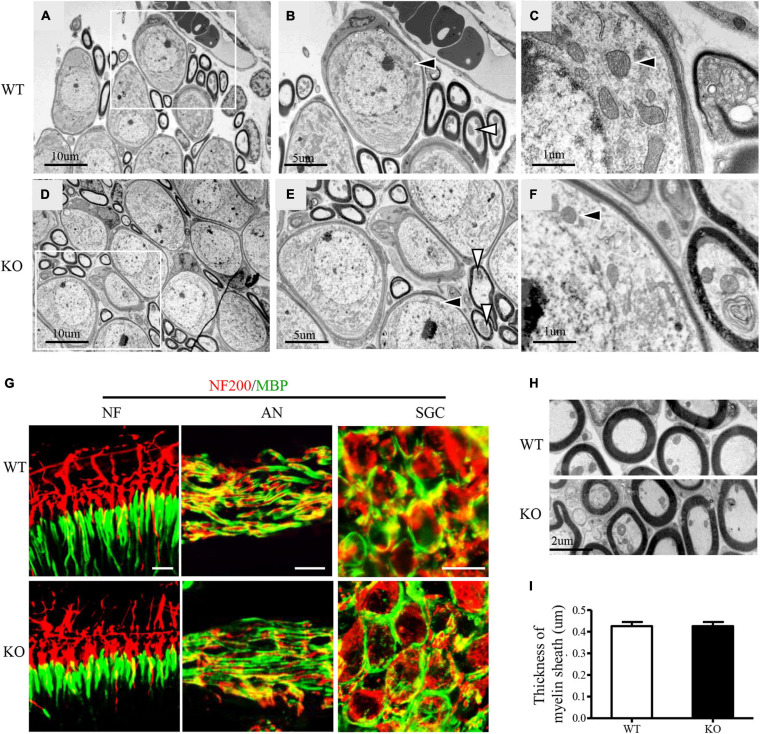
Subcellular structures of auditory nerve and SGCs in *C1ql1* WT and *C1ql1* KO mice. **(A–C)** The subcellular structures of auditory nerve and SGCs in *C1ql1* WT mice. **(B)** Inside the white box in **(A)** are auditory nerve (white arrow) and SGCs (black arrow), which are shown magnified. **(C)** Is a further magnification and mitochondria with normal morphology (black arrow) in SGC. **(D–F)** The subcellular structures of auditory nerve and SGCs in *C1ql1* KO mice. **(E)** Inside the white box in **(D)** are auditory nerve (white arrow) and SGCs (black arrow), which are shown magnified. **(F)** is a further magnification and mitochondria with normal morphology (black arrow) in SGC. **(G)** Immunofluorescence staining shows normal myelin sheath in the ending of auditory nerve fibers (left), in the middle part of auditory nerve fibers (middle), and in SGSs (right) of *C1ql1* WT mice and *C1ql1* KO mice, respectively. Myelin sheath was marked with anti-MBP antibody (green) and auditory nerve fibers with anti-NF200 (red). Scale bar = 10 μm. **(H)** Transmission electron microscopy shows myelin sheath of auditory nerve fibers in *C1ql1* WT mice and *C1ql1* KO mice. **(I)** Measured on electron micrographs, no significant difference was seen between the *C1ql1* WT mice group (*n* = 10) and *C1ql1* KO mice group (*n* = 10). Unpaired *t*-test, *P* = 0.22.

Further, we explored whether deficiency of C1QL1 protein affected spiral ganglion and auditory nerve myelin. To answer this question, we performed immunofluorescence staining and transmission electron microscopy. The ultrastructure of the SGCs of *C1ql1* KO mice looked similar to those of the wild type mice (Figures 5A–F). The number of SGNs of *C1ql1* KO mice was also not statically different from that of the wild-type mice (Figure 5G). We measured the thickness of the myelin sheath of the auditory nerve from the electron micrograph. The mean thickness of myelin sheath was 0.426 ± 0.060 μm in WT mice and 0.459 ± 0.056 μm in KO mice (Figures 5H,I). No significant difference was seen between the WT (*n* = 10) and KO mice (*P* = 0.22, *n* = 10). Our result suggests that loss of C1QL1 did not affect morphology of spiral ganglion and nerve myelin.

### Loss of C1QL1 Did Not Affect Morphology, Number, and Function of Cochlear Inner Hair Cells

**FIGURE 6:**
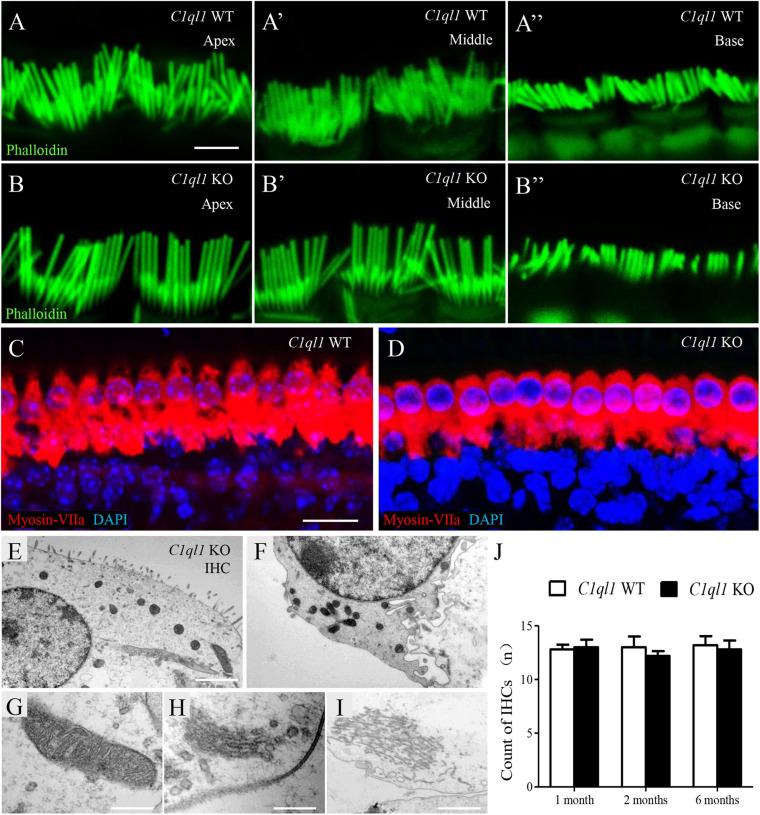
Morphology of cochlear inner hair cells in *C1ql1* WT mice and *C1ql1* KO mice. **(A,B)** The stereocilia in apex turn, middle turn and base turn of inner hair cells (green, labeled by phalloidin) in *C1ql1* WT mice and *C1ql1* KO mice. Scale bar = 2.5 μm. **(C)** The normal gross morphology of IHCs in *C1ql1* WT mice. **(D)** The gross morphology of IHCs in *C1ql1* KO mice. Cytoplasm of inner hair cells was marked with anti-myosin VIIa antibody (red) and nucleus of cells with DAPI (blue). Scale bar = 10 μm. **(E–I)** Subcellular structures of inner hair cells in *C1ql1* KO mice, normal mitochondria, Golgi apparatus, and endoplasmic reticulum were showed, respectively. Scale bar = 2.5 μm **(E)**. **(J)** Count of OHCs in *C1ql1* WT mice and *C1ql1* KO mice in postnatal 1 and 6 months.

**FIGURE 7:**
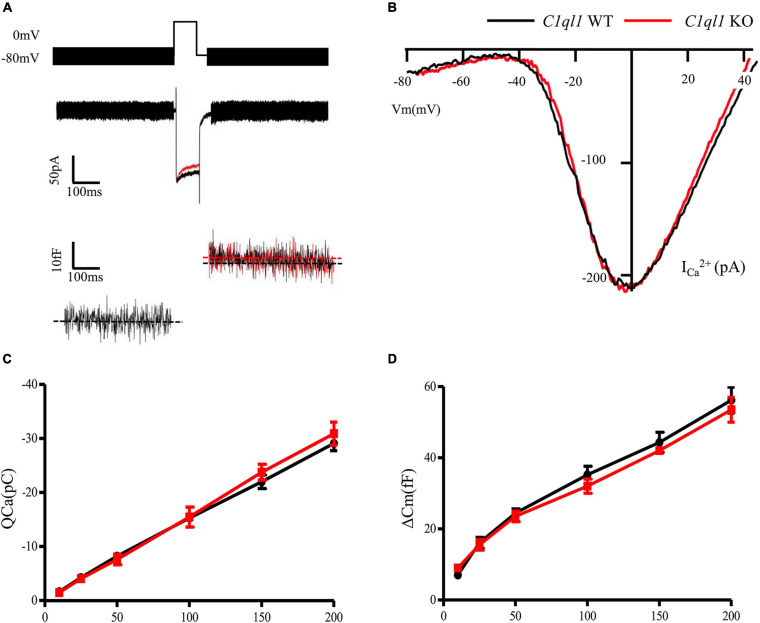
Function of cochlear inner hair cells in *C1ql1* WT mice and *C1ql1* KO mice. **(A)** The wave of potential changes in IHCs of *C1ql1* WT mice and *C1ql1* KO mice. **(B)** The IHCs in *C1ql1* KO mice also had normal biophysical properties. Ca++ current of IHCs seemed to be quite normal in *C1ql1* KO mice compare to WT mice. **(C)** Integral Ca++ current as a function of depolarization duration (in milliseconds). **(D)** Change in membrane capacitance as a function of depolarization duration (in milliseconds).

To determine if loss of C1QL1 affect inner hair cell (IHC) morphology, survival and function, immunofluorescence, transmission electron microscopy, and patch clamp techniques were used. The IHCs of the *C1ql1* KO mice have normal gross morphology, such as complete cell body and normal-looking stereocilia bundles (Figure 6). We counted the number of IHCs in the wild type and *C1ql1* KO mice in cochlea. No significant IHC loss was seen in the KO mice (*P* = 0.27, *n* = 5). To determine if C1QL1 is necessary for the formation of ribbon synapses and neurotransmitter release, voltage-dependent Ca^++^ current and change in membrane capacitance associated with release of neurotransmitter (endocytosis) were measured. The magnitude of voltage-dependent Ca^++^ current and membrane capacitance change were similar to those seem in wildtype IHCs (*n* = 3, *p* > 0.05) (Figure 7). Our results suggest the deficiency of C1QL1 did not affect the morphology, survival and synaptic transmission of IHCs.

### Loss of C1QL1 Leads to Progressive Loss of Outer Hair Cells

**FIGURE 8:**
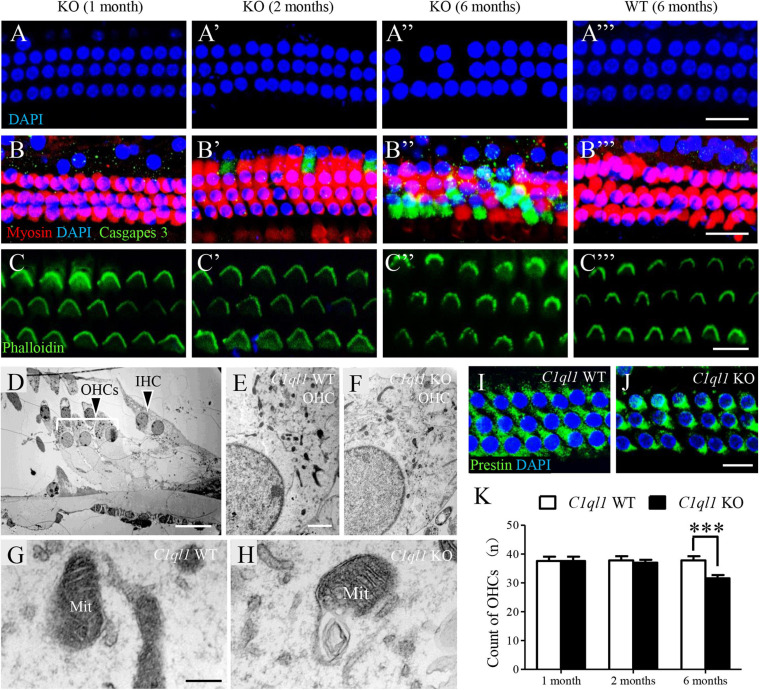
Morphology of cochlear outer hair cells in *C1ql1* WT mice and *C1ql1* KO mice. **(A–C)** Outer hair cells showed by immunofluorescence staining in *C1ql1* KO mice (postnatal 30 and 60 days, and 6 months, respectively) and in *C1ql1* WT mice (postnatal 6 months, as control group). Scale bar = 10 μm. **(A–A″′)** The nucleus (blue, labeled by DAPI) of outer hair cells in groups aforementioned. **(B–B″′)** The apoptotic signal (green, labeled by caspase 9) of outer hair cells in groups aforementioned. **(C–C″′)** The stereocilia of outer hair cells (green, labeled by phalloidin) in groups aforementioned. **(D)** Subcellular structures of outer hair cells in mice. Scale bar = 5 μm. **(E,F)** Much more mitochondria have been observed being swallowed by phagosomes in *C1ql1* KO mice than *C1ql1* WT mice. **(G,H)** Mitochondria in *C1ql1* WT mice and *C1ql1* KO mice. **(I,J)** Prestin in *C1ql1* WT mice and *C1ql1* KO mice. Scale bar = 10 μm. **(K)** Count of OHCs in *C1ql1* WT mice and *C1ql1* KO mice in postnatal 1 and 6 months. ^∗∗∗^*P* < 0.001.

We examined the morphology of OHCs by immunostaining and by transmission electron microscopy. Consistent with the results of DPOAE test, we found loss of OHCs in *C1ql1* KO mice after 2 months (Figures 8A′–C′). More OHC loss was observed at 6 months (Figures 8A′–C″). In contrast, no obvious loss of OHCs was seen in wild-type mice at the same age (Figures 8A″′–C″′). Interestingly, although remaining OHCs appeared to be normal with normal looking stereocilia bundles under light microscope, scanning electron microscopy showed more phagosomes in the cytoplasm of OHCs in *C1ql1* KO mice than in WT mice (Figures 8D–F). Besides, many mitochondria showed some signs of swallowing (Figures 8G,H).

## Discussion

*C1ql1*, widely expressed in brain, is an important gene associated with maturation, regulation and maintenance of synapses in nervous system (Bérubé et al., 1999; Hunsberger et al., 2005; Glanzer et al., 2007; Iijima et al., 2010). *C1ql1* is expressed in the central auditory pathways such as the ventral cochlear nucleus, lateral thalamus and trapezoid nuclei (Iijima et al., 2010). In the adult mouse cochlea, cell type-specific transcriptome analysis showed that *C1ql1* is expressed in IHCs, OHCs, pillar cells, and Deiters’ cells with differential expression in OHCs (Liu et al., 2018). But its function in hair cells remained unclear. Using loss of function approach in the mouse model, we showed that *C1ql1* was necessary for development and maintenance of innervations in IHCs and OHCs. Loss of C1QL1 also led to accelerated OHC loss. Taken together, our study suggests that *C1ql1* is indispensable to maintain normal auditory function.

We first used immunostaining to show that C1QL1 was expressed in hair cells. The protein expression is consistent with *C1ql1* expression detected in RNA-seq analysis (Liu et al., 2014; Li et al., 2018). Furthermore, we also detected its expression in the cytoplasm of spiral ganglion cells. This is also consistent with a previous study which showed that *C1ql1* is expressed in the mouse cochlear spiral ganglion neurons (Lu et al., 2011). We observed progressive hearing loss in *C1ql1* null mice. The hearing loss is likely caused by reduction of afferent innervation to IHCs and loss of OHCs. This was reflected by the elevation of ABR threshold, increased latency of ABR wave I, elevation of DPOAE threshold and loss of OHCs. Interestingly, loss of function of C1QL1 apparently did not impact the development and survival of IHCs as IHCs appeared to be normal with no obvious loss at 1 and 6 months. Apparently, C1QL1 is also not essential for the development of ribbon synapses as calcium current and neurotransmitter release recorded from *C1ql1*-null IHCs were not different from those of wildtype IHCs. It is interesting that reduction in afferent innervation to IHCs did not affect formation of presynaptic structure despite of the fact that the number of afferent fibers were reduced. The fact that IHCs and presynaptic structures develop normally suggest that that functional development of IHCs does not depend on influence of innervation or C1QL1. This is consistent with some previous studies which showed that morphological and physiological development of hair cells is autonomous (He, 1997; He et al., 2001).

Although *C1ql1*-null mice displayed loss of innervation in both IHCs and OHCs, it is unclear why C1QL1 is necessary and how it interacts with nerve terminals for synaptogenesis. Since presynaptic structures in hair cells remained normal, it is likely that C1QL1 may guide outgrowth of afferent and efferent fibers to hair cells. Previous studies have shown that the signaling pathway formed by the secreted protein C1QL1 and the adhesion-GPCR BAI3 (brain angiogenesis inhibitor 3) regulates the development of proper excitatory connectivity on cerebellar Purkinje cells (Sigoillot et al., 2015). C1QL1 controls climbing fiber synaptogenesis and territory on Purkinje cells and C1QL1’s modulation of Purkinje cell spinogenesis is BAI3-dependent. RNA-seq analysis shows that *Bai3* (*Baiap3*) is expressed in both IHCs and OHCs (Li et al., 2018) as well as in spiral ganglion neurons (Lu et al., 2011).

We observed reduction of fibers innervating OHCs in *C1ql1* KO mice. It is not clear if the reduction was only limited to type II fibers or if type II and efferent fibers to OHCs were both affected. Most nerve fibers crossing the tunnel are efferent fibers from MOC (Maison et al., 2016; Webber et al., 2021). We did not label these fibers using specific markers. However, a recent study showed that conditional deletion of *C1ql*1 only in OHCs reduced OHC afferent synapse maintenance (Biswas et al., 2021). We evaluated OHC function in *C1ql1* KO mice by measuring DPOAE. We observed a gradual decrease of DPOAE magnitude starting from 8 weeks after birth, which was correlated with the beginning of loss/degeneration of OHCs. Thus, our study suggests that long-term survival of OHCs depends on C1QL1 function. This is consistent with the fact that *C1ql1* is highly expressed in adult OHCs (Liu et al., 2014; Li et al., 2018). The cause of OHC loss is unknown. But it is unlikely due to reduction of afferent and/or efferent innervation as previous studies showed that denervation of efferent innervation did not impact hair cell survival. We note that a recent study showed that OHC-specific deletion of *C1ql1* did not reveal a compelling auditory phenotype (Biswas et al., 2021). We would like to point out that this is different from our study where *C1ql1* was unquietly deleted in hair cells and neurons. The auditory phenotype we observed was due to loss of innervations in IHCs and OHCs as well as loss of OHCs.

Loss of function due to mutations or deletion of many genes can lead to hearing loss with different auditory phenotypes. For example, mutations of *Tmc1* can lead to profound hearing loss due to loss of mechanotranduction in both IHCs and OHCs (Marcotti et al., 2006). Loss of *Slc26a5* can lead to loss of OHC motility and cochlear amplification (Liberman et al., 2002; Dallos et al., 2008). Loss of genes related to hair cell development can lead abnormal development of hair cells, resulting in hearing loss. Unless loss of function only affects IHC or neurons which is often manifested as auditory neuropath, the phenotype observed in loss of function of most genes is often mixed, as both IHCs and OHCs are affected. Deletion of *Slc26a5* also leads to unexpected IHC loss (Liberman et al., 2002; Dallos et al., 2008). The phenotype observed in *C1ql1* KO mice is due to loss of neuronal transmission (reflected by reduction in ABR threshold and increased latency in ABR wave I) and loss of OHC function (reflected by reduction in DPOAE magnitude).

Mutations or deficiencies affecting approximately 123 genes have been linked to inherited non-syndromic hearing loss in humans. There have been no reports of hearing loss due to mutations of *C1ql1*. It remains to be determined if *C1ql1* is a deafness-related gene. Since deletion of this gene can cause hearing loss, *C1ql1* mutations perhaps should be considered and screened in future genetic testing.

## Data Availability Statement

The original contributions presented in the study are included in the article/supplementary material, further inquiries can be directed to the corresponding author/s.

## Ethics Statement

The animal study was reviewed and approved by Animal Care Committee of the Capital Medical University.

## Author Contributions

YQ, WX, SY, ZD, TQ, LH, WW, and LZ were responsible for the experiment design. YQ, KL, and YL were responsible for data analysis. DH and SG were responsible for providing overall ideas. All authors contributed to the article and approved the submitted version.

## Conflict of Interest

The authors declare that the research was conducted in the absence of any commercial or financial relationships that could be construed as a potential conflict of interest.

## Publisher’s Note

All claims expressed in this article are solely those of the authors and do not necessarily represent those of their affiliated organizations, or those of the publisher, the editors and the reviewers. Any product that may be evaluated in this article, or claim that may be made by its manufacturer, is not guaranteed or endorsed by the publisher.
